# Stingray Envenomation Resulting in Neuropathy and Complex Regional Pain Syndrome Treated With Peripheral Nerve Stimulation: A Case Report

**DOI:** 10.7759/cureus.98242

**Published:** 2025-12-01

**Authors:** Ryan M Scott, Brian C McLean

**Affiliations:** 1 Anesthesiology/Pain Medicine, Naval Medical Center San Diego, San Diego, USA; 2 Anesthesiology/Pain Medicine, Pain Management Center, Naval Medical Center San Diego, San Diego, USA

**Keywords:** chronic and acute pain management, complex regional pain syndrome stages, mono-neuropathy, peripheral nerve stimulator, toxicology and envenomation

## Abstract

Stingray stings frequently result in severe but temporary pain; they can occasionally result in significant complications, including peripheral nerve injuries and complex regional pain syndrome (CRPS). CRPS is characterized by severe, persistent pain and functional impairment, often following a nerve injury. Conservative treatments may be insufficient, necessitating advanced management strategies.

A 19-year-old patient presented with radial neuropathy and CRPS following a stingray sting. Despite conservative treatment and orthopedic surgery, the patient's condition progressed, resulting in debilitating pain and functional limitations. The decision was made to place a SPRINT peripheral nerve stimulation (PNS) system (SPR, Cleveland, OH, US). Sixty days of right radial nerve stimulation resulted in a significant reduction in pain, improved function, and enhanced quality of life. The patient experienced sustained pain relief and functional improvement over the follow-up period after removal of the device.

While implanted spinal cord stimulators and permanent peripheral nerve stimulators have been used in the management of the pain from complex regional pain syndrome for many years, and this system is likely being used in the treatment of CRPS currently, this report adds to the literature evidence that use of a 60 day temporary PNS system early in the disease course may resolve the condition before it can become chronic. This case highlights the potential of PNS in managing complex pain syndromes and suggests that further research is needed to confirm its broader applicability and long-term benefits.

## Introduction

Stingray stings are a common marine injury, especially in Southern California, and can lead to a variety of complications due to the venomous nature of the sting and physical trauma inflicted by the stingray's barbed tail [1]. Stingray venom is composed of a complex mixture of proteins, enzymes, and other bioactive compounds that can cause intense pain, local tissue necrosis, and systemic reactions. Common immediate effects include severe pain, erythema, and edema [2]. Long-term complications can include chronic pain, infections, and, in rare cases, neurological damage due to direct trauma or envenomation effects [1]. The intense pain exhibited by stingray envenomation may be largely due to the presence of serotonin as well as two enzymes, 5-nucleotidase and phosphodiesterase [3].

Neurological complications from stingray stings can manifest as peripheral nerve injuries, which may result in complex regional pain syndrome (CRPS), previously known as reflex sympathetic dystrophy (RSD) [3]. CRPS is a debilitating condition characterized by persistent pain, usually following an injury. Symptoms include severe pain, swelling, changes in skin color and temperature, and functional impairment. The exact mechanisms of the pathophysiology of CRPS are not entirely understood, but are thought to involve an abnormal response of the nervous system to injury, with both peripheral, sympathetic, and central components contributing to the condition [4].

Management of neuropathic pain and CRPS involves a multidisciplinary approach. Initial treatment typically includes analgesics, anti-inflammatory medications, and topical medications. However, in cases where these methods are insufficient, more advanced therapies may be required. Options include physical therapy, occupational therapy, psychological support, and interventional procedures such as nerve blocks and spinal cord stimulation [4]. Advancements since at least the 1990s have explored peripheral nerve stimulators (PNS) as an effective method for managing neuropathic pain and CRPS; these stimulators were initially surgically implanted [4]. More recently we have seen the development of percutaneous implantable PNS, with promising results in treating many pain conditions, including CRPS [5,6] The SPRINT PNS system (SPR, Cleveland, OH, US) is unique in that it is not a permanent implant but can deliver therapy for up to 60 days on label and, despite the temporary nature, has shown promise in providing significant prolonged pain relief and improving function in patients with chronic pain syndromes [7].

## Case presentation

A 19-year-old healthy male patient suffered a stingray injury while trying to take a small stingray off his fishing hook. The patient received at least two injuries, one to the dorsal third finger and one to the volar medial wrist. He had immediate severe pain and swelling in the area with reduced range of motion of his hand and fingers. He removed the barbs himself with pliers and went home to clean the wound and ice it. When the pain and swelling did not subside after the first 48 hours, the patient presented to his primary care physician and was then directed to the Emergency Room (ER) for evaluation. In the ER, the patient was evaluated by the orthopedics team regarding an increased amount of contracture noted in the affected hand. Upon evaluation, the treating physicians thought he did not have an infection or urgent need for surgery. They recommended conservative care with splinting, occupational therapy, and pain management.

Upon presentation to our pain management clinic approximately three weeks later, the patient was noted to have continued neuropathic pain, swelling, sudomotor and trophic changes, and was diagnosed with ulnar and radial neuropathy and complex regional pain syndrome type II [8]. He had pain in the entire hand in the distribution of the ulnar, median, and radial nerves, with the most pain being in the radial nerve distribution. The next day, the patient underwent a cervical sympathetic nerve block, which only provided slight relief. Next, a right radial nerve block was performed, which resulted in almost complete relief of the patient’s pain.

**Figure 1:**
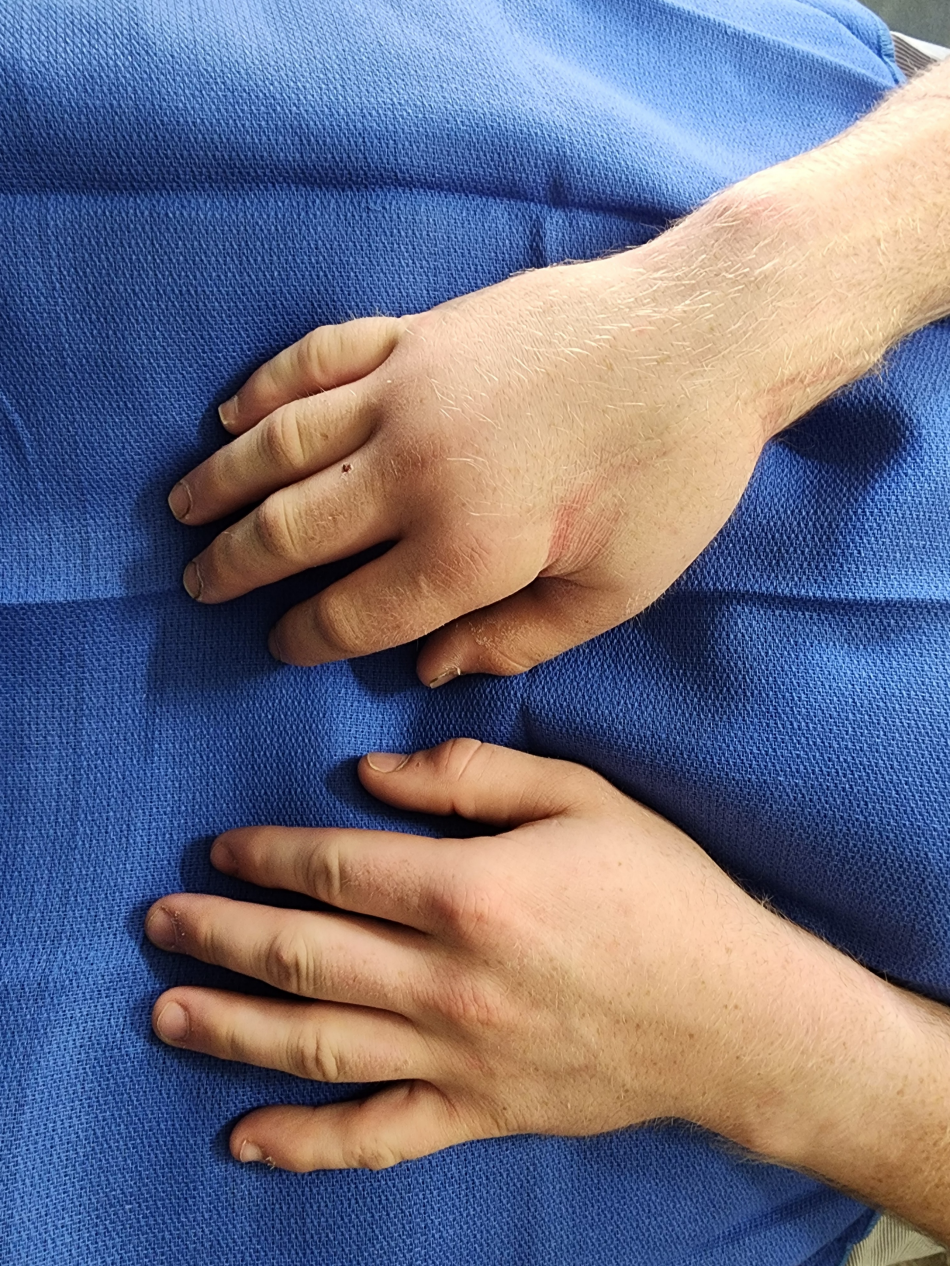
Affected hand vs non-affected hand with edema and trophic changes exhibited

There remained evidence of motor nerve injury with significant swelling, and so the orthopedic hand specialists were consulted again. An MRI of the affected hand showed no retained foreign body, but diffuse soft tissue edema with no clear fluid collection. The decision was made to explore the wound to evaluate for a structural cause of ongoing injury. The surgery was unrevealing for infection or retained barbs. Post-surgery, the previous chronic pain, edema, and trophic changes remained. The changes are seen in Figure 1.

The patient was counseled and offered treatment with placement of a peripheral nerve stimulator lead. A 60-day SPRINT PNS was placed under ultrasound guidance, targeting the radial nerve above the elbow. Stimulation covered the patient's greatest pain area. At the 2-week follow-up, the patient reported 70% pain relief while he continued to work with occupational therapy. At the 60-day visit, the patients’ pain had completely resolved, as had the edema, sudomotor, and trophic changes. He still had some atrophy and motor weakness of the thenar eminence, but denied any associated pain.

## Discussion

Stingray envenomation can lead to severe and complex complications, including CRPS, a devastating syndrome that remains a management challenge for clinicians across several disciplines. There continue to be barriers to the elucidation of the mechanisms that contribute to the development of the syndrome in every individual, which underscores the difficulties with finding adequate treatments [4].

The mainstay of initial treatment of stingray envenomation remains hot water immersion therapy and prudent antibiotic prophylaxis [1]. Rarely, a sting can lead to long-term sequelae, especially if not treated properly and expediently following the initial insult. Clinical studies have found stingray venom to induce neurogenic and inflammatory mediators as well as hyperalgesia [9]. There are few reports in the literature exploring subacute and chronic pain conditions resulting from stingray envenomation [10]. Despite the treatments recommended above, in rare cases of retained venom and/or barb material, there is an increased risk for continued insult leading to prolonged pain [11].

There is moderate evidence for the use of spinal cord stimulators (SCS) for the treatment of CRPS [12] to improve patients' pain and quality of life, but it remains to be shown if it has a significant effect on signs/symptoms of CRPS, opioid intake, and psychological facets of the syndrome [13]. This therapy remains expensive and can be lengthy to navigate the processes for trial and approval for implantation. It also requires a potentially permanent implant, which may not be a desirable option for several patients.

Peripheral nerve stimulation, such as the SPRINT PNS system, offers a promising approach for managing chronic pain and improving function in affected patients. The temporary nature of the system is well-suited for young, active patients and those who desire to avoid long-term or permanent implants. Mechanisms of PNS likely work through both peripheral and central pathways, with the gate theory of peripheral neuromodulation remaining a foundational paradigm for understanding the changes in ectopic discharges of nociceptive fibers [14]. PNS also downregulates and changes the local chemical milieu [15] and modulates several cortical and ascending/descending pathways [16], which points to a much more complex effect than the simplicity the gate theory might suggest.

There is a growing body of evidence for longer-term relief after the discontinuation of peripheral nerve stimulation. Sustained pain relief of up to 12 months from post-amputation pain has been demonstrated in a small randomized controlled trial using temporary PNS [7]. Previous case series and reports have provided evidence of effective CRPS treatment with temporary peripheral nerve stimulation; long-term success has been noted for up to 34 months after device removal [17]. In the 2022 Clinical Guidelines published by the American Society of Pain and Neuroscience, CRPS is a listed indication for the use of peripheral nerve stimulation. It is a Level 3, Grade C recommendation given the paucity of evidence to support [18].

## Conclusions

We presented a patient with early onset of CRPS, in whom we were able to implant a temporary PNS with complete resolution of pain; this may have helped him avoid years of pain and disability, as well as intense resource utilization to manage. This case highlights the potential efficacy of peripheral nerve stimulation in treating radial neuropathy and CRPS secondary to stingray envenomation. The positive outcome suggests that PNS can be a valuable addition to the treatment arsenal for neuropathic pain and CRPS, particularly when conventional methods are inadequate. Further research is warranted to establish the broader applicability and long-term benefits of PNS in similar cases.

## References

[1] Clark RF, Girard RH, Rao D, Ly BT, Davis DP (2007). Stingray envenomation: a retrospective review of clinical presentation and treatment in 119 cases. J Emerg Med.

[2] Auerbach PS (1991). Marine envenomations. N Engl J Med.

[3] Kline A (2008). Stingray envenomation of the foot: a case report. Foot Ankle.

[4] Shim H, Rose J, Halle S, Shekane P (2019). Complex regional pain syndrome: a narrative review for the practising clinician. Br J Anaesth.

[5] Chmiela MA, Hendrickson M, Hale J, Liang C, Telefus P, Sagir A, Stanton-Hicks M (2021). Direct peripheral nerve stimulation for the treatment of complex regional pain syndrome: a 30-year review. Neuromodulation.

[6] Herschkowitz D, Kubias J (2019). A case report of wireless peripheral nerve stimulation for complex regional pain syndrome type-I of the upper extremity: 1 year follow up. Scand J Pain.

[7] Gilmore CA, Ilfeld BM, Rosenow JM (2020). Percutaneous 60-day peripheral nerve stimulation implant provides sustained relief of chronic pain following amputation: 12-month follow-up of a randomized, double-blind, placebo-controlled trial. Reg Anesth Pain Med.

[8] Harden NR, Bruehl S, Perez RS (2010). Validation of proposed diagnostic criteria (the "Budapest Criteria") for complex regional pain syndrome. Pain.

[9] Kimura LF, Santos-Neto M, Barbaro KC, Picolo G (2018). Potamotrygon motoro stingray venom induces both neurogenic and inflammatory pain behavior in rodents. Toxicon.

[10] Trickett R, Whitaker IS, Boyce DE (2009). Sting-ray injuries to the hand: case report, literature review and a suggested algorithm for management. J Plast Reconstr Aesthet Surg.

[11] Meyer PK (1997). Stingray injuries. Wilderness Environ Med.

[12] Sayed D (2021). Abstracts from the North American Neuromodulation Society's 2021 virtual meeting, January 15-16, 2021. Neuromodulation.

[13] Visnjevac O, Costandi S, Patel BA, Azer G, Agarwal P, Bolash R, Mekhail NA (2017). A comprehensive outcome-specific review of the use of spinal cord stimulation for complex regional pain syndrome. Pain Pract.

[14] Ong Sio LC, Hom B, Garg S, Abd-Elsayed A (2023). Mechanism of action of peripheral nerve stimulation for chronic pain: a narrative review. Int J Mol Sci.

[15] Papuć E, Rejdak K (2013). The role of neurostimulation in the treatment of neuropathic pain. Ann Agric Environ Med.

[16] Meyer-Frießem CH, Wiegand T, Eitner L, Maier C, Mainka T, Vollert J, Enax-Krumova EK (2019). Effects of spinal cord and peripheral nerve stimulation reflected in sensory profiles and endogenous pain modulation. Clin J Pain.

[17] Gutierrez GJ, Zurn CA, Crosby ND (2024). Sustained relief of complex regional pain syndrome (CRPS) pain following a 60-day peripheral nerve stimulation: a report of three cases. Cureus.

[18] Strand N, D'Souza RS, Hagedorn JM (2022). Evidence-based clinical guidelines from the American Society of Pain and Neuroscience for the use of implantable peripheral nerve stimulation in the treatment of chronic pain. J Pain Res.

